# Prevalence of unipolar mania in bipolar I disorder: a systematic review and meta-analysis of observational studies

**DOI:** 10.1017/S2045796026100791

**Published:** 2026-07-17

**Authors:** Francesco Bartoli, Daniele Cavaleri, Martina Citton, Giorgio Cucchi, Martina Monti, Aldo De Pietra, Cristina Crocamo, Giuseppe Carrà

**Affiliations:** School of Medicine and Surgery, University of Milano-Bicocca, Monza, Italy

**Keywords:** bipolar I disorder, manic episode, meta-analysis, prevalence, systematic review, unipolar mania

## Abstract

**Aims:**

Unipolar mania (UM), defined by the occurrence of manic episodes without a history of depression, is a topic of debate within the classification of affective disorders. However, its epidemiological burden remains unclear. This systematic review and meta-analysis aimed to estimate the prevalence of UM among individuals with bipolar type I disorder (BD-I) while exploring potential sources of heterogeneity.

**Methods:**

The study protocol was registered in Open Science Framework on 27 March 2025. Embase, MEDLINE and APA PsycInfo were searched. We included observational studies reporting data on UM prevalence rates in adults with BD-I. Pooled prevalence was estimated using the Freeman–Tukey double arcsine transformation, employing a restricted maximum likelihood random-effects model. Subgroup and meta-regression analyses were implemented to explore sources of heterogeneity.

**Results:**

We included 26 studies, encompassing 35 independent samples and 17,716 individuals with BD-I. The pooled prevalence of UM was 21.1% (95% confidence interval: 15.5–27.4%). Although potential publication bias was detected (Egger’s *p* = 0.010), the trim-and-fill method did not impute any missing studies. No differences were found between clinical and community-based studies (*p* = 0.966). However, prevalence estimates were influenced by both geographical area (*p* = 0.020) and study quality (*p* = 0.014). Rate differences across studies may also be attributable to variations in UM diagnostic definition.

**Conclusions:**

People with UM represent a significant subset of BD-I cases worldwide, warranting greater clinical awareness. The observed rate variability emphasizes the impact of sociocultural and methodological factors on UM diagnosis. Further research is necessary to refine diagnostic criteria and evaluate optimal treatment approaches for individuals with UM.

## Introduction

In recent years, the diagnostic concept of unipolar mania (UM) has involved a growing scientific debate (Yazıcı, 2014; Angst and Grobler, 2015; Bartoli, 2023). People with this condition experience the typical symptoms of mania, including elevated mood, grandiosity, pressured speech, decreased need of sleep and hypersexuality (Kendler, 2017), without any history of major depressive episodes (Angst *et al.*, 2019). Even though UM was originally described more than 40 years ago (Abrams *et al.*, 1979; Nurnberger *et al.*, 1979; Pfohl *et al.*, 1982), current diagnostic systems – such as the Diagnostic and Statistical Manual of Mental Disorders (American Psychiatric Association, 2022) and the International Classification of Diseases (World Health Organization, 2022) – do not explicitly include this nosological entity. Whereas bipolar II disorder (BD-II) requires both depressive and hypomanic episodes, bipolar I disorder (BD-I) can be diagnosed on the basis of a single manic episode. Accordingly, UM should be classified within the nosological category of BD-I. However, including a disorder that, by definition, is unipolar, such as UM, within the category of disorders characterized by a bipolar course may appear counterintuitive and difficult to justify. Consistently, it has been suggested that UM can be a separate diagnostic entity (Angst, 2015; Angst and Grobler, 2015). Indeed, the recent Clinical Research Diagnostic Criteria for Bipolar Illness (Ghaemi *et al.*, 2022) propose that some patients might experience manic episodes only, without any history of depression, considering UM as an independent condition. Moreover, a recent international expert consensus has recognized UM as a potential clinical phenotype to be considered alongside the standard subtypes of BD (McIntyre *et al.*, 2022). In light of this, a recent meta-analysis has proposed the nosological independence of UM, showing that people with UM may significantly differ from those with manic-depressive BD: they are more often males, with an earlier age at onset and showing higher rates of hyperthymic temperamental traits, psychotic features and hospital admissions (Bartoli *et al.*, 2023a).

However, little is known about the epidemiological burden of UM, specifically the proportion of people with BD-I who have never experienced depressive episodes. Current epidemiological data rely on sparse community-based and clinical studies. For instance, the US National Epidemiologic Survey on Alcohol and Related Conditions reported a prevalence of UM among people with BD comprised between 5% and 7%, depending on the criteria used (Baek *et al.*, 2014), while significantly lower rates have been observed in studies from the United Kingdom (∼1%) and France (∼3%) (Stokes *et al.*, 2020). Conversely, clinical studies from low- and middle-income countries have shown markedly higher prevalence rates, suggesting that UM may represent nearly a half, or even more, out of people with BD-I (Rangappa *et al.*, 2016; Amamou *et al.*, 2018).

Nonetheless, no systematic synthesis of worldwide epidemiological data on UM is available so far. To shed light on this topic, we performed a systematic review and meta-analysis aimed at estimating the rates of UM in people with BD-I, while accounting for possible sources of heterogeneity, including geographical area of origin, sample characteristics, quality of included studies and the UM definition used.

## Method

### Study design and protocol

The current systematic review and meta-analysis is based on the Meta-analyses Of Observational Studies in Epidemiology (MOOSE) Reporting Guidelines (Brooke *et al.*, 2021). The study protocol registration was completed in the Open Science Framework registries on 27 March 2025 (doi: 10.17605/OSF.IO/K95HFj). The MOOSE checklist is reported in the Supplementary Table 1.

### Eligibility criteria

We included any observational studies providing data on UM prevalence rates in adults with BD-I. We excluded studies based on samples with a mean age <18 years and those that also included people with BD-II, BD not otherwise specified, and/or schizoaffective disorder without providing information on the subsample with BD-I. We excluded studies published before the release date of DSM-IV (American Psychiatric Association, 1994), as well as those using data from another study based on the same sample, to avoid duplicated results (Lunny *et al.*, 2021). We also excluded scientific reports not undergoing the peer-review process, such as conference abstracts, dissertations and grey literature.

### Search strategy and study selection

We searched Embase, MEDLINE (via Ovid) and APA PsycInfo databases (via EBSCOhost) for articles indexed from 1994 to 31 March 2025, without language restrictions. Query strings (including keywords ‘unipolar’, ‘monopolar’, ‘pure’, ‘mania’, ‘manic’ and ‘bipolar’) were adapted for each database. The full search strategy is reported in Supplementary Table 2. We carried out an additional, post hoc, non-systematic search on Google Scholar to check whether further studies were retrievable, as well as a manual search of the reference lists of a recent review (Bartoli *et al.*, 2023a). In case additional information or clarification was needed, we planned to contact the corresponding authors of potentially eligible articles at a later stage.

Once the preliminary screening based on titles and abstracts was completed, full texts were retrieved to assess studies according to inclusion criteria for final eligibility. The screening process was completed by three investigators (M.C., G.Cu. and M.M.) independently, and reasons for exclusion after full-text review were recorded. Disagreements concerning suitability for inclusion were resolved by discussion and consensus involving all authors.

### Data extraction

We used a standard .xlsx template to extract key information for all eligible studies: year of publication; country; recruitment source; type of sample (clinical or community-based); inclusion criteria; sample size, mean age and sex; illness duration; and assessment methods, definition and proportion of UM. Data extraction was performed starting on 14 April 2025. Three authors (F.B., C.C. and A.D.P.) independently extracted data for a blind check of accuracy.

### Quality assessment

We conducted a quality assessment of the included studies, deriving items from available tools according to the PERSyst recommendations (Migliavaca *et al.*, 2020a). Specifically, we evaluated: i) study representativeness, ii) sample size adequacy and iii) the quality of diagnostic assessment. A study was considered representative if data on the target population were retrieved from community-based samples or from large clinical data sources. The sample size was considered adequate, based on existing evidence (Baek *et al.*, 2014; Manchia *et al.*, 2025), if the study included at least 385 participants with BD-I, ensuring a margin of error of ±3% around an expected prevalence of 10%, at a 95% confidence level. Studies with smaller sample sizes were judged at higher risk of imprecision. Finally, the accuracy of BD-I diagnosis was ascertained by assessing whether standardized diagnostic interviews, such as the Structured Clinical Interview for DSM, the Composite International Diagnostic Interview or the Mini International Neuropsychiatric Interview, had been used. Studies meeting at least two of the three previously mentioned criteria were considered to be of higher quality compared to others.

### Data analysis

To estimate UM prevalence rates in BD-I with the corresponding 95% confidence intervals (CIs), we applied the Freeman–Tukey double arcsine transformation. We pooled data using the restricted maximum likelihood random-effects model. For studies providing multiple proportion values of UM according to different definitions, we followed a conservative approach, retaining the lower value for inclusion in the meta-analysis. Publication bias was assessed using Egger’s linear regression test (Page *et al.*, 2021). Where statistical significance was observed, the trim-and-fill method was applied (Duval and Tweedie, 2000).

**Figure 1.**
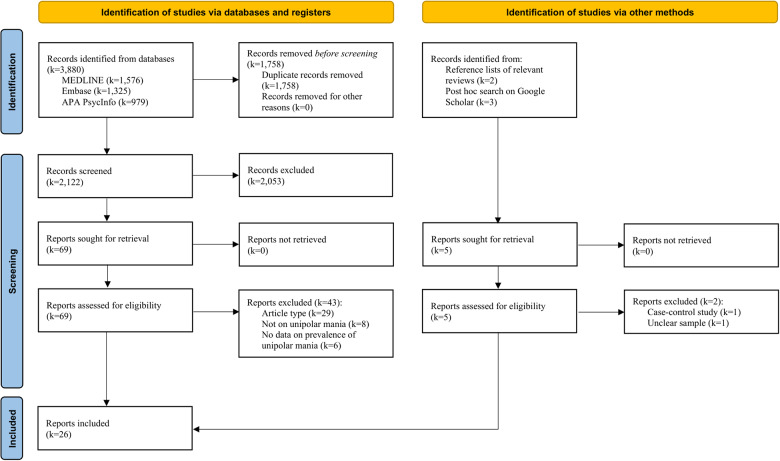
Flow chart of study inclusion process. Figure 1 long description.

Moreover, to deal with the expected skewed distribution of prevalence rates, we also reported the overall median along with lower (Q1) and upper (Q3) quartiles of the UM prevalence rates for descriptive purposes. Considering the generally low consistency of meta-analyses of prevalence rates (*I*^2^ > 90%) (Migliavaca *et al.*, 2020b), we did not use standard *I*^2^ statistics to measure heterogeneity across studies (Higgins *et al.*, 2003), considering that a high *I*^2^ value in a meta-analysis of prevalence may not be discriminative of high heterogeneity (Migliavaca *et al.*, 2022). Instead, to test the potential inconsistency across studies, we explored variations in UM prevalence rates based on specific study characteristics. Firstly, we ran random-effects meta-regression analyses testing the possible moderating effect of continuous variables from the sample, such as mean age, proportion of men and average disease duration. Secondly, we conducted subgroup analyses exploring differences in UM rates by the recruitment setting (clinical vs. community-based samples), the quality (high vs. low) and the geographical area of included studies. Subgroup differences were tested by estimating the relevant *p*-values from meta-regression analyses. Moreover, we conducted supplementary meta-analyses based on the UM definitions specifically used in each study, considering i) the number of required manic episodes (1, 2, 3 or 4) and ii) the time to diagnosis, i.e., the minimum length of illness duration required to establishing the UM condition (none or minimal vs. at least 4–5 years vs. at least 10 years or more). Data analyses were performed using Stata statistical software, Release 19 (StataCorp LLC, 2025, College Station, TX).

## Results

### Study selection

Our systematic search generated 3,880 records, namely 1,576 from Embase, 1,325 from MEDLINE and 979 from APA PsycInfo. After deduplication, there were 2,122 papers left to be screened, including 5 additional articles, 3 retrieved from Google Scholar and 2 from the reference list of a relevant review (Bartoli *et al.*, 2023a). Following screening by titles and abstracts, 69 studies were identified as potentially eligible. The final screening of full texts identified 26 studies, comprising 35 independent samples, which met the eligibility criteria for inclusion in the meta-analysis (Shulman and Tohen, 1994; Kirov and Murray, 1999; Aghanwa, 2001; Yazici *et al.*, 2002; Solomon *et al.*, 2003; Perugi *et al.*, 2007; Dakhlaoui *et al.*, 2008; Beesdo *et al.*, 2009; Andrade-Nascimento *et al.*, 2011; Akarsu *et al.*, 2012; Douki *et al.*, 2012; Yazıcı and Cakır, 2012; Baek *et al.*, 2014; Grobler *et al.*, 2014; Rajkumar, 2016; Rangappa *et al.*, 2016; Subramanian *et al.*, 2016; Amamou *et al.*, 2018; Angst *et al.*, 2019; Stokes *et al.*, 2020; Gorgulu *et al.*, 2021; Grover *et al.*, 2021; Adiukwu *et al.*, 2023; Chang *et al.*, 2023; Wikström *et al.*, 2023; Manchia *et al.*, 2025).

A flowchart with details of screening, study selection process and reasons for exclusion is presented in Figure 1. A list of the articles excluded after full-text review with relative reasons for exclusion is reported in Supplementary Table 3.

### Study characteristics

Included studies were published between 1994 (Shulman and Tohen, 1994) and 2025 (Manchia *et al.*, 2025). Most studies were written in English, two in French (Dakhlaoui *et al.*, 2008; Douki *et al.*, 2012) and one in Turkish (Akarsu *et al.*, 2012). Four studies (Douki *et al.*, 2012; Angst *et al.*, 2019; Stokes *et al.*, 2020; Chang *et al.*, 2023) included two or more independent samples that were used as single study units in this meta-analysis. Of the 35 independent samples, 14 were from a European country, 7 from Asia, 6 from Africa, 4 from North America, 3 from South America (all from Brazil) and 1 from Oceania (Fiji Islands). Moreover, 23 were based on clinical samples and 10 on community-based data, while 2 used mixed clinical and community samples (the NESDA cohort [Angst *et al.*, 2019] and the GREAT cohort [Chang *et al.*, 2023]). The study characteristics are reported in Table 1.

**Table 1.** Characteristics of the included studies Table 1 long description.

Author(s), year *Cohort*	Country	Setting	BD-I sample size	Age (years) *mean ± SD*	Male *n* (*%*)	Duration of illness *mean ± SD*	UM definition^a^	UM patients *n* (*%*)
Adiukwu *et al.*, 2023	Nigeria	Clinical	116	–	59 (51.3)	–	≥2 manic episodes without depressive episodes	29 (25.0)
Aghanwa, 2001	Fiji	Clinical	108	–	–	–	≥3 manic episodes without depressive episodes for at least 4 years	51 (47.2)
Akarsu *et al.*, 2012	Turkey	Clinical	108	31.0 ± 11.8^b^	85 (78.7)	7.5 ± 7.5^b^	≥4 manic episodes without depressive episodes for at least 10 years	16 (14.8)
Amamou *et al.*, 2018	Tunisia	Clinical	173	43.3 ± 12.2	118 (68.2)	17.9 ± 10.2	≥3 manic episodes without depressive episodes	98 (56.6)
Andrade-Nascimento *et al.*, 2011	Brazil	Clinical	298	41.6 ± 12.1^b^	95 (31.9)	15.6^b^^,^^c^^,^^d^	≥1 manic episode without depressive episodes for at least 15 years	16 (5.4)
Angst *et al.*, 2019 *Zurich Study*^e^	Switzerland	Community	37	49.5^c^	17 (45.9)	–	≥1 manic episode without depressive episodes	18 (48.6)
Angst *et al.*, 2019 *ZInEP Survey*^e^	Switzerland	Community	11	25.8^c^	5 (45.5)	–	≥1 manic episode without depressive episodes	4 (36.4)
Angst *et al.*, 2019 *Sao Paulo Survey*^e^	Brazil	Community	35	38.7^c^	17 (48.6)	–	≥1 manic episode without depressive episodes	6 (17.1)
Angst *et al.*, 2019 *Pelotas Study*^e^	Brazil	Community	117	20.2^c^	42 (35.9)	–	≥1 manic episode without depressive episodes	37 (31.6)
Angst *et al.*, 2019 *NCS-R*^e^	USA	Community	101	35.8^c^	38 (37.6)	–	≥1 manic episode without depressive episodes	33 (32.7)
Angst *et al.*, 2019 *CoLaus/PsyCoLaus Study*^e^	Switzerland	Community	45	52.0^c^	29 (64.4)	–	≥1 manic episode without depressive episodes	9 (20.0)
Angst *et al.*, 2019 *NESDA*^e^	Netherlands	Mixed	86	40.9^c^	38 (44.2)	–	≥1 manic episode without depressive episodes	2 (2.3)
Baek *et al.*, 2014	USA	Community	1,411	–	–	–	≥3 manic episodes without depressive episodes for at least 10 years	76 (5.4)
Beesdo *et al.*, 2009	Germany	Community	84	–	42 (50.0)	–	≥1 manic episode without depressive episodes	33 (39.3)
Chang *et al.*, 2023 *GREAT database*^e^	Taiwan	Mixed	1,015	40.0 ± 13.1^b^	456 (44.9)	11.6 ± 10.1^d^	≥1 manic episode without depressive episodes	131 (12.9)
Chang *et al.*, 2023 *PIMC dataset*^e^	Taiwan	Clinical	8,343	38.0 ± 15.3^b^	3,963 (47.5)	–	≥1 manic episode without depressive episodes	1,241 (14.9)
Dakhlaoui *et al.*, 2008	Tunisia	Clinical	72	36.2^c^	42 (58.3)	11.6^c^	≥2 manic episodes without depressive episodes	47 (65.3)
Douki *et al.*, 2012 *French sample*^e^	France	Clinical	40	44.6^c^	–	15.2^c^^,^^d^	≥1 manic episode without depressive episodes	5 (12.5)
Douki *et al.*, 2012 *Tunisian sample*^e^	Tunisia	Clinical	40	40.8^c^	–	14.8^c^^,^^d^	≥1 manic episode without depressive episodes	16 (40.0)
Gorgulu *et al.*, 2021	Turkey	Clinical	495	–	–	–	≥4 manic episodes without depressive episodes for at least 4 years	40 (8.1)
Grobler *et al.*, 2014	South Africa	Clinical	94	–	–	–	≥3 manic episodes without depressive episodes	42 (44.7)
Grover *et al.*, 2021	India	Clinical	714	45.6 ± 10.4	–	–	≥4 manic episodes without depressive episodes for at least 5 years	42 (5.9)
Kirov and Murray, 1999	UK	Clinical	105	37.8 ± 8.8^b^	44 (41.9)	14.1 ± 9.2 ^b^	≥1 manic episode without depressive episodes	15 (14.3)
Manchia *et al.*, 2025	Italy	Clinical	649	–	–	–	≥1 manic episode without depressive episodes	59 (9.1)
Perugi *et al.*, 2007	Italy	Clinical	155	–	–	–	≥3 manic episodes without depressive episodes for at least 10 years	19 (12.3)
Rajkumar, 2016	India	Clinical	84	–	–	–	≥3 manic episodes without depressive episodes	21 (25.0)
Rangappa *et al.*, 2016	India	Clinical	285	33.6 ± 11.7	162 (56.8)	11.5^c^^,^^d^	≥3 manic episodes without depressive episodes for at least 5 years	137 (48.1)
Shulman and Tohen, 1994	USA	Clinical	50	–	–	9.8^c^	≥3 manic episodes without depressive episodes for at least 10 years	6 (12.0)
Solomon *et al.*, 2003	USA	Clinical	163	–	–	–	≥1 manic episode without depressive episodes	14 (8.6)
Stokes *et al.*, 2020 *South London cohort*^e^	UK	Community	1,458	54.3 ± 14.5	569 (39.0)	–	≥3 manic episodes without depressive episodes for at least 10 years	17 (1.2)
Stokes *et al.*, 2020 *French cohort*^e^	France	Clinical	392	41.1 ± 13.3	180 (45.9)	16.1 ± 10.9	≥3 manic episodes without depressive episodes for at least 10 years	13 (3.3)
Subramanian *et al.*, 2016	India	Clinical	150	37.8 ± 9.8	72 (48.0)	13.4 ± 8.3	≥1 manic episode without depressive episodes	79 (52.7)
Wikström *et al.*, 2023	Ethiopia	Community	289	–	–	–	≥3 manic episodes without depressive episodes	56 (19.4)
Yazıcı and Cakır, 2012	Turkey	Clinical	121	44.8 ± 1.2	48 (39.7)	18.2 ± 10.9	≥4 hypo/manic (at least 1 full manic) episodes without depressive episodes for at least 4 years	34 (28.1)
Yazici *et al.*, 2002	Turkey	Clinical	272	39.0 ± 13.2^b^	104 (38.2)	14.0 ± 8.6^b^	≥4 hypo/manic (at least 1 full manic) episodes without depressive episodes for at least 4 years	48 (17.6)

BD-I: bipolar type I disorder; GREAT: Genomic Research and Epidemiological Studies for Affective Disorders in Taiwan; NCS-R: National Comorbidity Survey Replication; NESDA: Netherlands Study of Depression and Anxiety; PIMC: Psychiatric Inpatients Medical Claim; SD: standard deviation; UK: United Kingdom; UM: unipolar mania; USA: United States of America; ZInEP: ZürcherImpulsprogrammzurnachhaltigenEntwicklung der Psychiatrie (Zurich Program for Sustainable Development of Mental Health Services).

a If multiple definitions were available, we reported the most stringent diagnostic criteria for unipolar mania.

b Estimated from subgroups means and SDs.

c SD not available.

d Approximated from current mean age and age at onset.

e Different cohorts related to the same paper.

According to the chosen quality assessment, including i) evaluation of study representativeness, ii) sample size adequacy and iii) BD-I diagnostic process, 18 samples were identified as being of high quality, meeting at least 2 of these criteria, while the remaining samples were classified as of low quality. The item-level quality assessment is reported in Supplementary Table 4.

### Prevalence of unipolar mania

**Figure 2.**
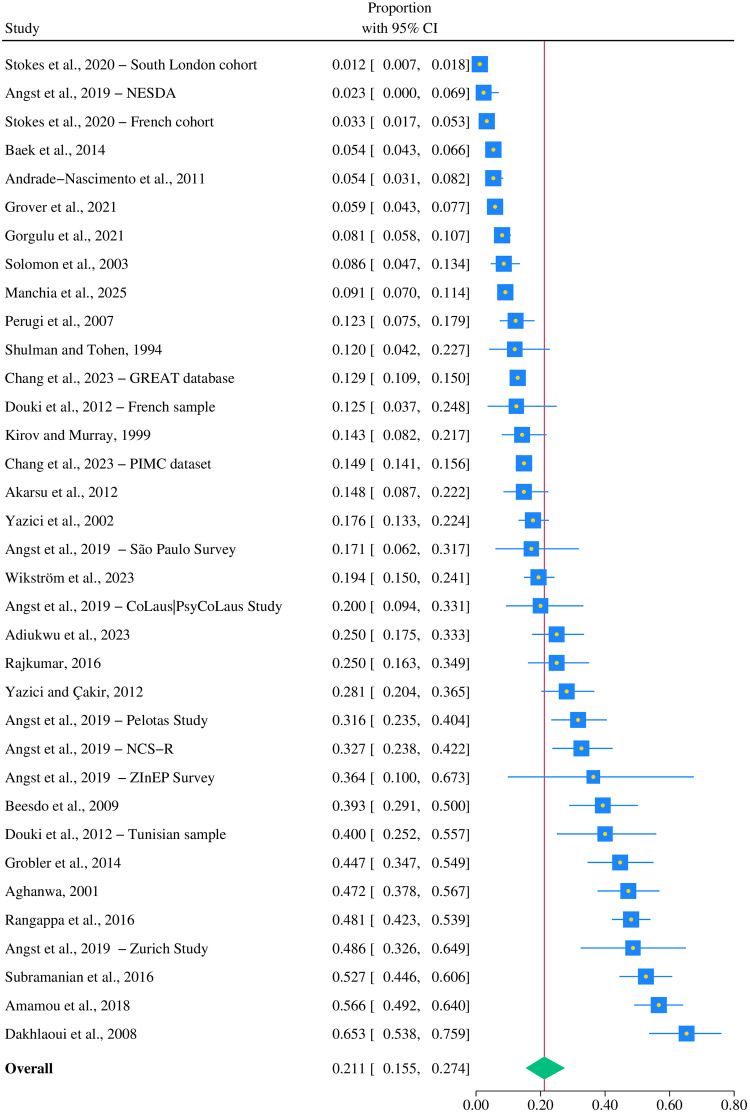
Forest plot of the meta-analysis of studies reporting the prevalence of unipolar mania in bipolar type I disorder. Figure 2 long description.

Data from 26 studies, including 35 independent samples accounting for a total of 17,716 individuals with BD-I, showed that the pooled prevalence of UM was 21.1% (95% CI: 15.5–27.4%). The relevant forest plot is shown in Figure 2. The median value was 17.6% (Q1–Q3: 10.6–37.8%). We estimated a potential publication bias by using Egger’s test (*z* = 2.59; *p* = 0.010). However, supplementary analyses using the trim-and-fill method did not impute any missing studies.

No moderating effects by study sample mean age (coeff.: −0.019; *p* = 0.140), proportion of males (coeff.: 0.014; *p* = 0.100) and average duration of illness (coeff.: 0.005; *p* = 0.922) were estimated by meta-regression analyses.

**Figure 3.**
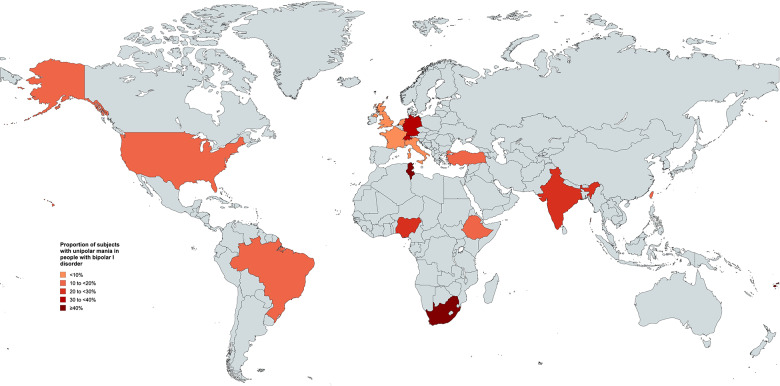
World map showing country-level rates of unipolar mania in bipolar type I disorder.

Subgroup analyses showed no differences in UM rates according to the type of sample (*p* = 0.966), being similar in clinical (22.4%; 95% CI: 15.2–30.5%) and community samples (21.8%; 95% CI: 11.6–33.9%) (Supplementary Figure 1). However, both geographical area (*p* = 0.020) and quality of included studies (*p* = 0.014) influenced the prevalence estimates. For instance, both in Europe (15.2%; 95% CI: 8.5–23.4%) and in North America (13.0%; 95% CI: 4.0–26.0%), the UM rates appeared to be markedly lower than in Africa (41.1%; 95% CI: 26.7–56.2%) (Supplementary Figure 2). Moreover, UM in BD-I was overrepresented in studies with low quality (28.8%; 95% CI: 20.2–38.1%) as compared with those with high quality (14.9%; 95% CI: 8.9–22.2%) (Supplementary Figure 3). The world map showing country-level rates of UM in BD-I is shown in Figure 3.

The results of subgroup analyses are reported in Table 2.

**Table 2.** Proportion of unipolar mania in bipolar type I disorder: subgroup analyses Table 2 long description.

Subgroup	*K*	*k*	*N*	PFT (95%CI)	*p*-Value^a^
*Type of sample*^b^					0.966
Clinical sample	23	24	13,027	22.4% (15.2–30.5%)	
Community-based sample	5	9	3,588	21.8% (11.6–33.9%)	
*Geographical area*^c^					**0.020**
Africa	6	6	784	41.1% (26.7–56.2%)	
Asia	6	7	11,086	21.6% (9.6–36.7%)	
Europe	10	14	3,563	15.2% (8.5–23.4%)	
North America	4	4	1,725	13.0% (4.0–26.0%)	
South America	2	3	450	16.3% (3.6–35.2%)	
*Quality*^d^					**0.014**
Higher quality	12	18	15,331	14.9% (8.9–22.2%)	
Lower quality	16	17	2,385	28.8% (20.2–38.1%)	

95% CI: 95% confidence interval; *K*: number of studies; *k*: number of subsamples; *N*: number of subjects; PFT: proportion based on Freeman–Tukey double arcsine transformation.

a Based on random-effects meta-regression analyses for subgroup differences (statistical significance reported in bold).

b Two studies based on mixed samples were not included.

c Oceania, for which there is only a single study (conducted in the Fiji Islands), is not included.

d Based on the number of quality items met.

Finally, prevalence estimates seemed to be dependent on the definition used for UM. Reported UM rates were lower in studies requiring more episodes (Supplementary Figures 4–7) and longer observation for the UM diagnosis (Supplementary Figures 8–10). Since some studies provided multiple prevalence estimates based on different definitions of UM, we were unable to conduct meta-regression analyses to assess subgroup differences.

The synthesis of findings by UM definition is reported in Table 3.

**Table 3.** Synthesis of findings by unipolar mania definition Table 3 long description.

Definition	*K*	*k*	*N*	PFT (95% CI)
*By minimum number of episodes*				
At least 1 episode	15	23	14,019	27.7 (19.3–36.9)
At least 2 episodes	5	5	1,275	36.3 (18.5–56.3)
At least 3 episodes	11	12	5,213	20.2 (9.9–33.0)
At least 4 episodes	6	6	3,121	12.3 (6.7–19.3)
*By duration of observation*				
None or minimal observation required	17	26	14,082	27.0 (19.8–34.9)
At least 4 or 5 years of observation required	8	8	3,514	19.8 (9.5–32.6)
At least 10 years of observation required	6	7	3,872	5.4 (2.7–8.9)

95% CI: 95% confidence interval; *K*: number of studies; *k*: number of subsamples; *N*: number of subjects; PFT: proportion based on Freeman–Tukey double arcsine transformation.

## Discussion

### Summary and interpretation of findings

To our knowledge, this is the first systematic review and meta-analysis aimed at assessing the prevalence rates of individuals with BD-I who have never been diagnosed with depression, i.e., those with a UM course. Based on data including almost 18,000 participants, we found that more than a fifth of subjects with BD-I worldwide experienced UM. This cumulative proportion is significantly higher than prior evidence, which suggested that UM might involve no more than 5–10% of individuals with BD-I (Baek *et al.*, 2014; McIntyre *et al.*, 2022). Our findings emphasize the epidemiological burden of this condition, which may be distinct from manic-depressive BD-I in terms of prognosis, treatment response and clinical features (Ghaemi *et al.*, 2022; Bartoli *et al.*, 2023a).

However, it is important to acknowledge that several factors are likely to influence the observed prevalence of UM. Firstly, our meta-analysis showed that there are remarkable geographical variations in prevalence rates. For instance, in Europe and the US, the proportion of UM appears to be lower, involving less than 20% of people with BD-I, while in Africa, rates are markedly higher, corresponding to around a half of subjects in samples from both Tunisia and South Africa. It is likely that UM in BD-I is much more common in non-Western countries (Angst and Grobler, 2015), which is consistent with pioneering data highlighting the possible role of ethnicity (Makanjuola, 1985; Kirov and Murray, 1999). In particular, since the early 1980s, it has been estimated that in Yoruba Nigerian patients with mania over a five-year period, more than 50% had recurrent UM, around 35% had a single manic episode, while only 10% of them had the typical manic-depressive course (Makanjuola, 1985). Moreover, individuals of African origin living in the UK were also more likely to present predominantly manic episodes than other people with BD-I (Kirov and Murray, 1999). In addition, Kennedy *et al.* (2004) showed that people of African-Caribbean and African descent were significantly less likely to have experienced a depressive episode before the manic onset, as compared to those of Caucasian ethnicity. While several hypotheses can be explored to explain differences in the proportion of UM among people with BD-I across countries and ethnicities, it is important to understand whether these may be related to contextual differences in the assessment of depression or, rather, to real differences in the presentation of affective symptoms (Kirov and Murray, 1999). Indeed, in contexts where access to care is limited (either because of reduced service availability or due to stigma), manic episodes may be more likely to be recognized than depressive episodes, particularly when the latter are not severe. Cultural factors may also contribute to cross-country differences by influencing the expression and regulation of affectivity (Haeri *et al.*, 2011), as well as the likelihood that these manifestations are captured by standard diagnostic instruments without proper contextualization (Gbadamosi *et al.*, 2022). In addition, stigma and fear of discrimination may reduce the likelihood of seeking treatment and of being recognized as depressed. Consistently, studies indicate that about one-third of people with mental illness in Africa experience some form of internalized stigma (Alemu *et al.*, 2023).

Secondly, from our findings, epidemiological figures tend to be markedly higher in studies with low quality. It is likely that issues related to poor representativeness, small sample size and an assessment not based on clinical interviews may have at least partially overestimated the proportion of UM in BD-I. In these studies, individuals with more severe clinical presentations and who are more likely to seek mental health services may be overrepresented. However, even when considering only UM rates from higher-quality studies, which were estimated to be around 15%, we can conclude that UM is not as rare as previously believed (Baek *et al.*, 2014; Stokes *et al.*, 2020).

Finally, prevalence rates seem to vary according to the definitions used for UM diagnosis. While some studies have defined UM based on a single manic episode without any depressive episode, others have emphasized the recurrent nature of this disorder. In addition, some studies reported a minimum period of observation to define a UM course in the absence of depression, while others did not use any temporal criterion. Thus, it is not surprising that, when more restrictive criteria concerning the number of manic episodes and the minimum length of observation required are used, the rates are significantly lower than in other studies. Possibly, before the nosological validity of UM can be claimed, several issues regarding its definition need to be resolved, more clearly identifying the criteria for this diagnosis. Moreover, it remains unclear how clinically meaningful symptoms of the opposite polarity may coexist in people with UM. For instance, it should be clarified whether only patients with pure mania should be considered in the UM category or also those with manic episodes with mixed features. This is a key issue, as the presence of mixed features, according to DSM-5 criteria, is a mood episode specifier and does not define mixed episodes (Bartoli *et al.*, 2020). Similarly, the role of mild, subthreshold depressive episodes remains uncertain. It is not yet determined whether these should be classified as UM or as an additional subtype of BD (Angst and Grobler, 2015). In addition, the presence of psychotic symptoms and the recurrent nature of mania could suggest a connection, and possibly overlap, between UM and schizoaffective disorder. Nonetheless, it is important to note that in individuals with schizoaffective disorder, psychotic symptoms occur independently of mood episodes (American Psychiatric Association, 2022). By contrast, in UM, psychotic features typically emerge only in the context of manic episodes and may arise directly from the euphoric affective state (Kendler, 2020). As a whole, the evidence deriving from our meta-analysis, demonstrating a high frequency of UM among people diagnosed with BD-I, along with previous findings showing that it is associated with specific clinical correlates (Bartoli *et al.*, 2023a), supports the hypothesis that UM is a condition that might be well differentiated from both manic-depressive BD and schizoaffective disorder.

### Clinical implications

Identifying UM may help group different phenotypes of the affective spectrum in a clearer and more homogeneous framework (Bartoli, 2023; Bartoli *et al.*, 2024). Accounting for UM, affective disorders could be classified following the same organization of sub-affective states, including the hyperthymic, dysthymic and cyclothymic temperaments (Ghaemi *et al.*, 2022), according to the different degree of depressive or manic predominance. These may include i) UM in which only manic episodes occur; ii) unipolar depression in which, conversely, there are only depressive episodes (the so-called major depressive disorder) and iii) the manic-depressive disorder (the conventional BD), classified according to its predominant polarity (Cavaleri *et al.*, 2025). However, a clear differentiation of UM within the context of the affective spectrum may also have possible implications for clinical practice. Even though there are no specific clinical trials focused on UM, it has been hypothesized that its pharmacological treatment could be less complex than for manic-depressive BD (Angst *et al.*, 2019). This is consistent with evidence showing that available pharmacological agents for BD are more effective in preventing mania than depression (Kishi *et al.*, 2021). Lithium, the gold standard treatment of BD-I, is likely to be essential in the treatment of UM, as it has proven to be more effective at preventing mania than depression (Fountoulakis *et al.*, 2022). However, this should be confirmed by future research since preliminary findings, though based on observational data, have counterintuitively suggested that lithium for UM may be less effective than for BD-I (Yazıcı, 2014). Additional research is also needed to clarify the potential role of antipsychotics in UM. It is likely that combining an antipsychotic with a mood stabilizer is necessary in most cases, given that people with UM are more prone to experiencing manic episodes with psychotic features (Bartoli *et al.*, 2023b). Moreover, they may be less susceptible to the depressogenic effects of some antipsychotics with antimanic properties (Goldberg, 2020). Considering that manic states are often associated with decreased insight and poor adherence to daily oral medications (Bulteau *et al.*, 2018; Ghosal *et al.*, 2021), long-acting injectable antipsychotics may also be considered a valid therapeutic option for UM (Bartoli *et al.*, 2023b). Another important clinical aspect is related to the sleep pattern, which may be regulated by both antipsychotics with sedative properties and a short-term treatment with benzodiazepines. A large UK study has recently demonstrated that people with UM had more disturbed sleep and increased difficulty getting up in the morning than subjects with a manic-depressive BD (Sangha *et al.*, 2022). In sum, further research is needed to examine the response of UM to conventional treatments currently used for BD-I. Tools capable of predicting the trajectories of manic episodes – whether occurring in the context of UM or a manic–depressive course – are needed to enable early initiation of appropriate and optimized treatment.

### Limitations

Although this work provides an extensive and systematic overview of UM prevalence, several limitations should be acknowledged. Firstly, any interpretation of the variations in UM rates among individuals with BD-I across different geographical areas should take into account that epidemiological data on UM are available from only a limited number of countries. Secondly, we should consider the limited statistical power of some included studies, as most involved small samples, typically fewer than 200 participants with BD-I. Thirdly, the retrospective assessment of UM based on past mood episodes may introduce recall bias in some studies, possibly influencing the results of our meta-analysis. In addition, although we accounted for certain methodological differences across studies, namely the required number of manic episodes and the time to diagnosis for UM as well as for sample characteristics, some of these analyses were based on a limited number of included studies, generating potentially imprecise estimates. Finally, as significant evidence of publication bias was detected, it is possible that studies estimating lower rates of UM remain unpublished. However, the absence of missing studies imputed by the trim-and-fill test suggests that this finding might be at least partly due to substantial between-study heterogeneity (Sterne *et al.*, 2000).

## Conclusions

Among individuals with BD-I, nearly a quarter have never been diagnosed with major depressive episodes. The substantial geographical heterogeneity in UM prevalence rates may suggest that ethnic, sociocultural and methodological variables are important for the diagnosis and presentation of UM. Overall, our findings support the need for further research on UM to better operationalize its conceptualization, standardize its diagnostic framework and improve its therapeutic management.

## Supporting information

10.1017/S2045796026100791.sm001 Bartoli et al. supplementary material Bartoli et al. supplementary material

## Data Availability

The data that support the findings of this systematic review, extracted from the included studies, are available from the corresponding author, F. Bartoli, upon reasonable request.

## Supplementary material

The supplementary material for this article can be found at https://doi.org/10.1017/S2045796026100791.

## Figure 1 Long description

The flowchart outlines the study inclusion process with two main sections: ′Identification of studies via databases and registers′ and ′Identification of studies via other methods′. In the first section, records identified from databases are listed: MEDLINE (k=1,576), Embase (k=1,325), APA PsycInfo (k=979). Records removed before screening include duplicates (k=1,758) and records removed for other reasons (k=0). Records screened total 2,122, with 2,053 excluded. Reports sought for retrieval are 69, with 0 not retrieved. Reports assessed for eligibility are 69, with 43 excluded for reasons such as wrong article type (k=29), not being on unipolar mania (k=8) and not reporting data on prevalence of unipolar mania (k=6) . In the second section, records identified from reference lists (k=2) or via Google Scholar (k=3) total 5. Reports sought for retrieval are 5, with 0 not retrieved. Reports assessed for eligibility are 5, with 2 excluded. In total, 26 reports were included.

Navigate back to Figure 1.

## Figure 2 Long description

Study A forest plot with a left column labeled Study and a right column labeled Proportion with 95 percent confidence interval. The plotted scale runs from 0.00 to 0.80 with tick labels at 0.00, 0.20, 0.40, 0.60 and 0.80. A vertical reference line is drawn at 0.20. Each study is shown as a square marker with a horizontal line for the 95 percent confidence interval, aligned to the proportion value listed in text. The studies and values shown are: Stokes et al., 2020 – South London cohort: 0.012 (0.007, 0.018) Angst et al., 2019 – NESDA: 0.023 (0.010, 0.069) Stokes et al., 2020 – French cohort: 0.033 (0.017, 0.053) Baek et al., 2014: 0.054 (0.043, 0.066) Andrade-Nascimento et al., 2011: 0.054 (0.031, 0.082) Grover et al., 2021: 0.059 (0.043, 0.077) Gorgulu et al., 2021: 0.081 (0.058, 0.107) Solomon et al., 2003: 0.086 (0.047, 0.136) Manchia et al., 2025: 0.091 (0.070, 0.114) Perugi et al., 2007: 0.123 (0.075, 0.179) Shulman and Tohen, 1994: 0.120 (0.042, 0.227) Chang et al., 2022 – GREAT database: 0.129 (0.109, 0.150) Douki et al., 2012 – French sample: 0.125 (0.037, 0.248) Kirov and Murray, 1999: 0.143 (0.082, 0.217) Chang et al., 2022 – PIMC dataset: 0.149 (0.141, 0.156) Akarsu et al., 2012: 0.148 (0.087, 0.222) Yazici et al., 2002: 0.176 (0.133, 0.224) Angst et al., 2019 – São Paulo Survey: 0.171 (0.062, 0.317) Wikström et al., 2022: 0.194 (0.150, 0.241) Angst et al., 2019 – CoLausPsyCoLaus Study: 0.200 (0.194, 0.331) Adikwu et al., 2023: 0.250 (0.175, 0.333) Rajkumar, 2016: 0.250 (0.163, 0.349) Yazici and Çakir, 2012: 0.281 (0.240, 0.365) Angst et al., 2019 – Pelotas Study: 0.316 (0.235, 0.404) Angst et al., 2019 – NCS-R: 0.327 (0.238, 0.422) Angst et al., 2019 – ZInEP Survey: 0.364 (0.100, 0.673) Besedo et al., 2009: 0.393 (0.291, 0.500) Douki et al., 2012 – Tunisian sample: 0.400 (0.252, 0.557) Grohler et al., 2014: 0.447 (0.347, 0.549) Aghamwa, 2001: 0.472 (0.378, 0.567) Rangappa et al., 2016: 0.481 (0.423, 0.539) Angst et al., 2019 – Zurich Study: 0.486 (0.326, 0.649) Subramanian et al., 2016: 0.527 (0.446, 0.609) Amamou et al., 2018: 0.566 (0.492, 0.640) Dakhlaoui et al., 2008: 0.653 (0.538, 0.759). At the bottom, overall is shown with a diamond marker and the value 0.211 (0.155, 0.274).

Navigate back to Figure 2.

## Table 1 Long description

The table compiles characteristics of 33 cohorts of people with bipolar I disorder across countries, reporting sample size, mean age, percent male, illness duration, how unipolar mania was defined, and how many met that definition. Definitions vary widely, from one lifetime manic episode with no depressive episodes to multiple manic or hypomanic episodes sustained over several years, which affects comparability of rates. Reported unipolar mania proportions span from 1.2% in a UK community cohort (South London) to 65.3% in a Tunisian clinical cohort (Dakhlaoui 2008), with other high values including 56.6% in Tunisia (Amamou 2018) and 52.7% in India (Subramanian 2016). Large datasets show lower to mid-range proportions, such as 14.9% in Taiwan’s PIMC clinical dataset (over 8,000 participants) and 12.9% in Taiwan’s GREAT mixed setting cohort (over 1,000 participants). Community surveys in the Angst 2019 series range from 2.3% in the Netherlands (NESDA) to 48.6% in Switzerland (Zurich Study), indicating substantial variation even within community settings. Where reported, mean ages range from about 20 years (Brazil Pelotas) to about 54 years (UK South London), and male percentages vary roughly from low 30s to high 70s across cohorts. Many cells are missing for age, sex, or illness duration, and some values are estimated or approximated, so cross-study comparisons should be interpreted cautiously.

Navigate back to Table 1.

## Table 2 Long description

The table reports pooled estimates of the proportion of unipolar mania among people with bipolar type I disorder, summarized across study subgroups with study counts, subsamples, and total participants. By sample type, estimates are similar: clinical samples 22.4% with a 95% confidence interval from 15.2% to 30.5%, and community-based samples 21.8% with a 95% confidence interval from 11.6% to 33.9%; the subgroup difference is not statistically significant. By geographical area, proportions differ and the subgroup test indicates a statistically significant difference: Africa 41.1% (26.7% to 56.2%), Asia 21.6% (9.6% to 36.7%), Europe 15.2% (8.5% to 23.4%), North America 13.0% (4.0% to 26.0%), and South America 16.3% (3.6% to 35.2%). By study quality, higher-quality studies report a lower pooled proportion at 14.9% (8.9% to 22.2%) than lower-quality studies at 28.8% (20.2% to 38.1%), and this difference is statistically significant. Confidence intervals are wide for some regions and smaller subgroups, so precision varies and comparisons should consider the differing numbers of studies and participants.

Navigate back to Table 2.

## Table 3 Long description

The table summarizes pooled proportions of unipolar mania across studies, grouped by how unipolar mania was defined, and reports study counts, subsamples, total participants, and pooled proportion with a 95 percent confidence interval. By minimum number of episodes, the pooled proportion is 27.7 percent for at least one episode (15 studies, 23 subsamples, 14,019 participants), 36.3 percent for at least two episodes (5 studies, 1,275 participants), 20.2 percent for at least three episodes (11 studies, 5,213 participants), and 12.3 percent for at least four episodes (6 studies, 3,121 participants). By duration of observation, the pooled proportion is 27.0 percent when none or minimal observation is required (17 studies, 26 subsamples, 14,082 participants), 19.8 percent when at least four or five years are required (8 studies, 3,514 participants), and 5.4 percent when at least ten years are required (6 studies, 7 subsamples, 3,872 participants). The clearest pattern is that stricter follow-up duration requirements correspond to markedly lower pooled proportions. Episode-count definitions do not show a steady stepwise change, with the highest estimate at the two-episode threshold and lower estimates as the minimum episode requirement increases further. Confidence intervals are wide for several groups, especially the two-episode definition, so differences should be interpreted cautiously.

Navigate back to Table 3.
