# Supplementary material for: Prevalence of unipolar mania in bipolar I disorder: a systematic review and meta-analysis of observational studies
Source: Epidemiol Psychiatr Sci. 2026 Jul 17;35:e38. doi: 10.1017/S2045796026100791 (PMC13420152; doi:10.1017/S2045796026100791)

Supplementary Table 1. MOOSE (Meta-analyses Of Observational Studies in Epidemiology) checklist.

Reporting Criteria	Reported (Yes/No)	Reported on Page No.
**Reporting of Background**		
Problem definition	Yes	1-2
Hypothesis statement	Yes	2
Description of Study Outcome(s)	Yes	2,5
Type of exposure or intervention used	Yes	2
Type of study design used	Yes	Title, Abstract, 2-4
Study population	Yes	2
**Reporting of Search Strategy**		
Qualifications of searchers (eg, librarians and investigators)	Yes	3
Search strategy, including time period included in the synthesis and keywords	Yes	3, Suppl.
Effort to include all available studies, including contact with authors	Yes	3
Databases and registries searched	Yes	3, Suppl.
Search software used, name and version, including special features used (eg, explosion)	Yes	3, Suppl.
Use of hand searching (eg, reference lists of obtained articles)	Yes	3
List of citations located and those excluded, including justification	Yes	6, Suppl
Method for addressing articles published in languages other than English	Yes	3,6
Method of handling abstracts and unpublished studies	Yes	3
Description of any contact with authors	Yes	3
**Reporting of Methods**		
Description of relevance or appropriateness of studies assembled for assessing the hypothesis to be tested	Yes	2-4
Rationale for the selection and coding of data (eg, sound clinical principles or convenience)	Yes	3,4
Documentation of how data were classified and coded (eg, multiple raters, blinding, and interrater reliability)	Yes	3,4
Assessment of confounding (eg, comparability of cases and controls in studies where appropriate	Yes	5

Reporting Criteria	Reported (Yes/No)	Reported on Page No.
Assessment of study quality, including blinding of quality assessors; stratification or regression on possible predictors of study results	Yes	4
Assessment of heterogeneity	Yes	5
Description of statistical methods (eg, complete description of fixed or random effects models, justification of whether the chosen models account for predictors of study results, dose-response models, or cumulative meta-analysis) in sufficient detail to be replicated	Yes	4,5
Provision of appropriate tables and graphics	Yes	Tab. 1-3, Fig. 1-3, Suppl. Material
**Reporting of Results**		
Table giving descriptive information for each study included	Yes	6, Table 1
Results of sensitivity testing (eg, subgroup analysis)	Yes	7,8
Indication of statistical uncertainty of findings	Yes	6-8
**Reporting of Discussion**		
Quantitative assessment of bias (eg, publication bias)	Yes	7,13
Justification for exclusion (eg, exclusion of non–English-language citations)	Yes	3
Assessment of quality of included studies	Yes	4,7,8,10
**Reporting of Conclusions**		
Consideration of alternative explanations for observed results	Yes	9-13
Generalization of the conclusions (ie, appropriate for the data presented and within the domain of the literature review)	Yes	11,13
Guidelines for future research	Yes	12,13
Disclosure of funding source	Yes	Funding Statement

Supplementary Table 2. Search strings by database.

**MEDLINE** (via Ovid)	"(unipolar OR monopolar OR pure).ti,ab AND (mania OR manic).ti,ab AND bipolar"
**Embase** (via Embase)	"(unipolar:ti,ab OR monopolar:ti,ab OR pure:ti,ab) AND (mania:ti,ab OR manic:ti,ab) AND bipolar"
**APA PsycInfo** (via EBSCOhost)	"(TI(unipolar OR monopolar OR pure) OR AB(unipolar OR monopolar OR pure)) AND (TI(mania OR manic) OR AB(mania OR manic)) AND bipolar"

Supplementary Table 3. List of the articles excluded after full text review with related reasons for exclusion.

**Records from the main database search excluded after full text review (k=43)**	
Excluded for article type (k=29)	1. Angst J, Grobler C. Unipolar mania: a necessary diagnostic concept. *Eur Arch Psychiatry Clin Neurosci*. 2015; **265(4)**:273–80.<br>2. Angst J. Will mania survive DSM-5 and ICD-11? *Int J Bipolar Disord*. 2015; **3(1)**.<br>3. Bartoli F, Malhi GS, Carrà G. Combining predominant polarity and affective spectrum concepts in bipolar disorder: towards a novel theoretical and clinical perspective. *Int J Bipolar Disord*. 2024; **12(1)**.<br>4. Bartoli F, Nasti C, Palpella D, Piacenti S, Di Lella ME, Mauro S, et al. Characterizing the clinical profile of mania without major depressive episodes: a systematic review and meta-analysis of factors associated with unipolar mania. *Psychol Med*. 2023; **53(15)**:7277–86.<br>5. Bartoli F, Nasti C, Palpella D, Piacenti S, Di Lella ME, Mauro S, et al. Clinical factors associated with unipolar mania: A systematic review and meta-analysis. *Eur Psychiatry*. 2023; **66(S1)**:S506–6.<br>6. Bartoli F. The Diagnostic Concept of Unipolar Mania. *J Nerv Ment Dis*. 2023; **211(11)**:811–3.<br>7. Chang CE, Kuo PH. Psychiatric comorbidities distinguish pure mania from depressive-mania in bipolar disorder. *Bipolar Disord*. 2017; **19**:89.<br>8. Dondé C, Lepetit A, Lavigne B. Faut-il annoncer un diagnostic de trouble bipolaire à un patient qui n'a jamais connu la dépression ? Une revue systématique actualisée sur la manie récurrente. *Presse Med*. 2019; **48(11)**:1306–18.<br>9. Erdem M, Akarsu S, Ünlü AG, Alper M, Karaman D, Ak M. 2834 – Comparison of clinical and the sociodemographic characteristics of bipolar patients according to the presence of a history of depressive episode. *Eur Psychiatry*. 2013; **28**:1.<br>10. Felipe-Vergara T, Green MJ, Cooper SA. Manía unipolar: un ineludible diagnóstico. Revisión de la literatura a propósito de un caso. *Rev Chil Neuro-Psiquiat*. 2021; **59(4)**:302–7.<br>11. Ghaffarinejad A, Mehdizadeh Zare Anari A, Mirghiasi A. 2083 – Prevalence of unipolar mania and evaluation of the characteristics in unipolar and bipolar mania in kerman (Iran). *Eur Psychiatry*. 2013; **28**:1.<br>12. Grant BF, Stinson FS, Hasin DS, Dawson DA, Chou SP, Ruan WJ, et al. Prevalence, Correlates, and Comorbidity of Bipolar I Disorder and Axis I and II Disorders. *J Clin Psychiatry*. 2005; **66(10)**:1205–15.<br>13. Grobler C. Unipolar mania: A distinct entity. *Bipolar Disord*. 2021; **28(S1)**:36.<br>14. Harish T, Grover S, Basu D. Recurrent Unipolar Mania: Does it Warrant a Separate Nosological Status? *Ger J Psychiatry*. 2005; **8(1)**:8–15.<br>15. Joffe RT, Young LT, MacQueen GM. A two-illness model of bipolar disorder 1. *Bipolar Disord*. 1999; **1(1)**:25–30.<br>16. Khodaeifar F, Gudarzi SS, Ghaffarinejad A, Javanbakht M, Mirghiasi A. Relative frequency of unipolar mania among hospitalized patients and its associate features: a multicenter study in Iran. *Int J Psychiatry Clin Pract*. 2013; **17**:24–5.<br>17. Khodaeifar F, Gudarzi SS, Mahmoodi V, Ghaffarinejad A, Mirghiasi A, Javanbakht M. Relative frequency of unipolar mania among hospitalized patients with bipolar mood disorder and its associate features: A multicenter study in Iran. *Bipolar Disord*. 2014; **16**:115.<br>18. Lee S, Joo Y, Kim H, Park S Clinical characteristics of patients with recurrent mania. *Bipolar Disord*. 2014; **16**:116.<br>19. Lopes R, Azevedo J, Curral R, Esteves M, Roma-Torres A. Revisiting Unipolar Mania: an entity forgotten? *Arq Med*. 2013; **27(4)**:154–7.<br>20. Martin D., Smith D. Unipolar mania: A discrete nosological entity within a broad bipolar spectrum? Information from the Bipolar Disorder Research Network Study. *Bipolar Disord*. 2013; **15**:115.<br>21. Mateen Y, Lally J, Bokhari SQ. Chronic, Unipolar, Treatment-Resistant Mania: A Case Report and Literature Review. *BJPsych Open*. 2022; **8(S1)**:S122–3.<br>22. Mehta S. Unipolar Mania: Recent Updates and Review of the Literature. *Psychiatry J*. 2014; **2014**:1–6.<br>23. Rakesh G, Hatti S, Thirthahalli J, Reddy YCJ. 245 Predominance of Manic Episodes in the Course of Bipolar Disorder. *Asian J Psychiatry*. 2011;**4**:S48–9.

	24. Rybakowski JK. Painting "Mania." *J Affect Disord*. 2011; **128(3)**:319–20.<br>25. Schmitt A, Falkai P. Classification and neurobiological concepts of mania, bipolar disorder and major depression. *Eur Arch Psychiatry Clin Neurosci*. 2015; **265(4)**:271–2.<br>26. Sushma BR, Munivenkatappa S, Narayanaswamy JC, Jain S, Reddy YCJ. Predominant mania course in Indian patients with bipolar I disorder. *Asian J Psychiatry*. 2016 Aug; **22**:22–7.<br>27. Thirthalli J., Rakesh G., Hatti, SV., Reddy YCJ. Predominance of manic episodes in the course of bipolar disorder in India. *Bipolar Disord*. 2011; **22**:13.<br>28. Yazıcı O. Unipolar mania: A distinct entity? *J Affect Disord*. 2014; **152-154**:52–6.<br>29. Yüksel FV, Kurt A, Tüzer V, Göka E. Unipolar Mani. *Klinik Psikiyatri* 2004; **7**:161–6.
Excluded for not being on unipolar mania (k=8)	1. Goldberg JF, Garno JL, Portera L, Leon AC, Kocsis JH. Qualitative differences in manic symptoms during mixed versus pure mania. *Compr Psychiatry*. 2000; **41(4)**:237–41.<br>2. McGinty KR, Janos J, Seay J, Youngstrom JK, Findling RL, Youngstrom EA, et al. Comparing Self-Reported Quality of Life in Youth with Bipolar Versus Other Disorders. *Bipolar Disord*. 2023; **25**:648–60.<br>3. Prakash O, Channaveerachari Naveen Kumar, Prafulla Shivakumar, Bharath S, Varghese M. Clinical presentation of mania compared with depression: data from a geriatric clinic in India. *Int Psychogeriatr*. 2009; **21(4)**:764–7.<br>4. Rosa AR, Andreazza AC, Kunz M, Gomes F, Santin A, Sanchez-Moreno J, et al. Predominant polarity in bipolar disorder: diagnostic implications. *J Affect Disord*. 2008; **107(1-3)**:45–51.<br>5. Rossi A, Enrico Daneluzzo, Arduini L, Domenico MD, Stratta P, Petruzzi C. Cognitive symptoms of mania in pure and mixed episodes evaluated with the Positive and Negative Syndrome Scale. *Eur Arch Psychiatry Clin Neurosci*. 2000; **250(5)**:254–6.<br>6. Serretti A, Olgiati P. Profiles of "manic" symptoms in bipolar I, bipolar II and major depressive disorders. *J Affect Disord*. 2005; **84(2-3)**:159–66.<br>7. Shah S, Aich T, Sandip Subedi. A factor analytical study report on mania from Nepal. *Indian J Psychiatry*. 2017; **59(2)**:196–6.<br>8. Shoaib AM, Dilsaver SC. Panic disorder in subjects with pure mania and depressive mania. *Anxiety*. 1994; **1(6)**:302–4.
Excluded for not having data on the prevalence of unipolar mania (k=6)	1. Angst J, Gerber-Werder R, Zuberbuhler HU, Gamma A. Is bipolar I disorder heterogeneous? *Eur Arch Psychiatry Clin Neurosci*. 2004; **254(2)**:82–91.<br>2. Khan O, Youssef NA. A brief history of polarity in mood and its diagnostic evolution. *Ann Clin Psychiatry*. 2018; **30(1)**:61–6.<br>3. Mittal PK, Mehta S, Solanki RK, Swami MK, Meena PS. A comparative study of seasonality and chronotype in unipolar mania vs. bipolar affective disorder. *Ger J Psychiatry*. 2013; **16(4)**:124–9.<br>4. Sangha N, Lyall LM, Cullen B, Whalley HC, Wyse CA, Smith DJ. Investigating the nosological status of unipolar mania within UK Biobank using objective and subjective measures of rest and activity. *Sleep Med*. 2022; **100**:S215–6.<br>5. Shi HM, Jiang DG. Unipolar mania over the course of a 15-year follow-up study. *Psychiatry Res*. 2025; **344**:116348.<br>6. Sonkurt HO, Altınöz AE, Danışman Sonkurt M, Köşger F. A distinct neurocognitive profile: unipolar mania. *Nord J Psychiatry*. 2022; **76(5)**:358–64.
**Records from Google Scholar additional search excluded after full text review (k=2)**	
Excluded for article type (k=1)	1. Amithabh S., Kini Ganesh, Kakunje Anil. Comparison of socio-demographic and clinical factors between "unipolar mania" and bipolar affective disorder. *Kerala J Psychiatry*. 2021; **34(2)**:135–41.
Excluded because of unclear sample (k=1)	1. Negash A. Alem A., Kebede D., Deyessa N., Shibre T., Kullgren G. Prevalence and clinical characteristics of bipolar I disorder in Butajira, Ethiopia: A community-based study. *J Affect Disord*. 2005; **87**:193–201.

Supplementary Table 4. Quality assessment of the included studies.

Study	Representativeness	Sample size	Assessment	Quality items §
Adiukwu et al., 2023	+	–	–	**1**
Aghanwa, 2001	+	–	–	**1**
Akarsu et al., 2012	–	–	–	**0**
Amamou et al., 2018	–	–	–	**0**
Andrade-Nascimento et al., 2011	+	–	+	**2**
Angst et al., 2019 – Zurich Study	+	–	+	**2**
Angst et al., 2019 – ZInEP Survey	+	–	–	**1**
Angst et al., 2019 – São Paulo Survey	+	–	+	**2**
Angst et al., 2019 – Pelotas Study	+	–	+	**2**
Angst et al., 2019 – NCS-R	+	–	+	**2**
Angst et al., 2019 – CoLaus|PsyCoLaus Study	+	–	+	**2**
Angst et al., 2019 – NESDA	+	–	+	**2**
Baek et al., 2014	+	+	+	**3**
Beesdo et al., 2009	+	–	+	**2**
Chang et al., 2022 – GREAT database	+	+	+	**3**
Chang et al., 2022 – PIMC dataset	+	+	–	**2**
Dakhlaoui et al., 2008	–	–	–	**0**
Douki et al., 2012 – French sample	–	–	–	**0**
Douki et al., 2012 – Tunisian sample	–	–	–	**0**
Gorgulu et al., 2021	–	+	–	**1**
Grobler et al., 2014	+	–	+	**2**
Grover et al., 2021	+	+	+	**3**
Kirov and Murray, 1999	–	–	–	**0**
Manchia et al., 2025	+	+	+	**3**
Perugi et al., 2007	–	–	+	**1**
Rajkumar, 2016	–	–	+	**1**
Rangappa et al., 2016	–	–	+	**1**
Shulman and Tohen, 1994	–	–	–	**0**
Solomon et al., 2003	+	–	+	**2**
Stokes et al., 2020 – South London cohort	+	+	–	**2**
Stokes et al., 2020 – French cohort	+	+	–	**2**
Subramanian et al., 2016	–	–	+	**1**
Wikström et al., 2022	+	–	+	**2**
Yazici and Çakir, 2012	–	–	+	**1**
Yazici et al., 2002	–	–	+	**1**

§ Number of quality items met.
Abbreviations: **GREAT**: Genomic Research and Epidemiological Studies for Affective Disorders in Taiwan; **NCS-R**: National Comorbidity Survey Replication; **NESDA**: Netherlands Study of Depression and Anxiety; **PIMC**: Psychiatric Inpatients Medical Claim; **ZInEP**: Zürcher Impulsprogramm zur nachhaltigen Entwicklung der Psychiatrie.

Supplementary Figure 1. Subgroup analysis according to the type of sample (clinical vs. community samples).

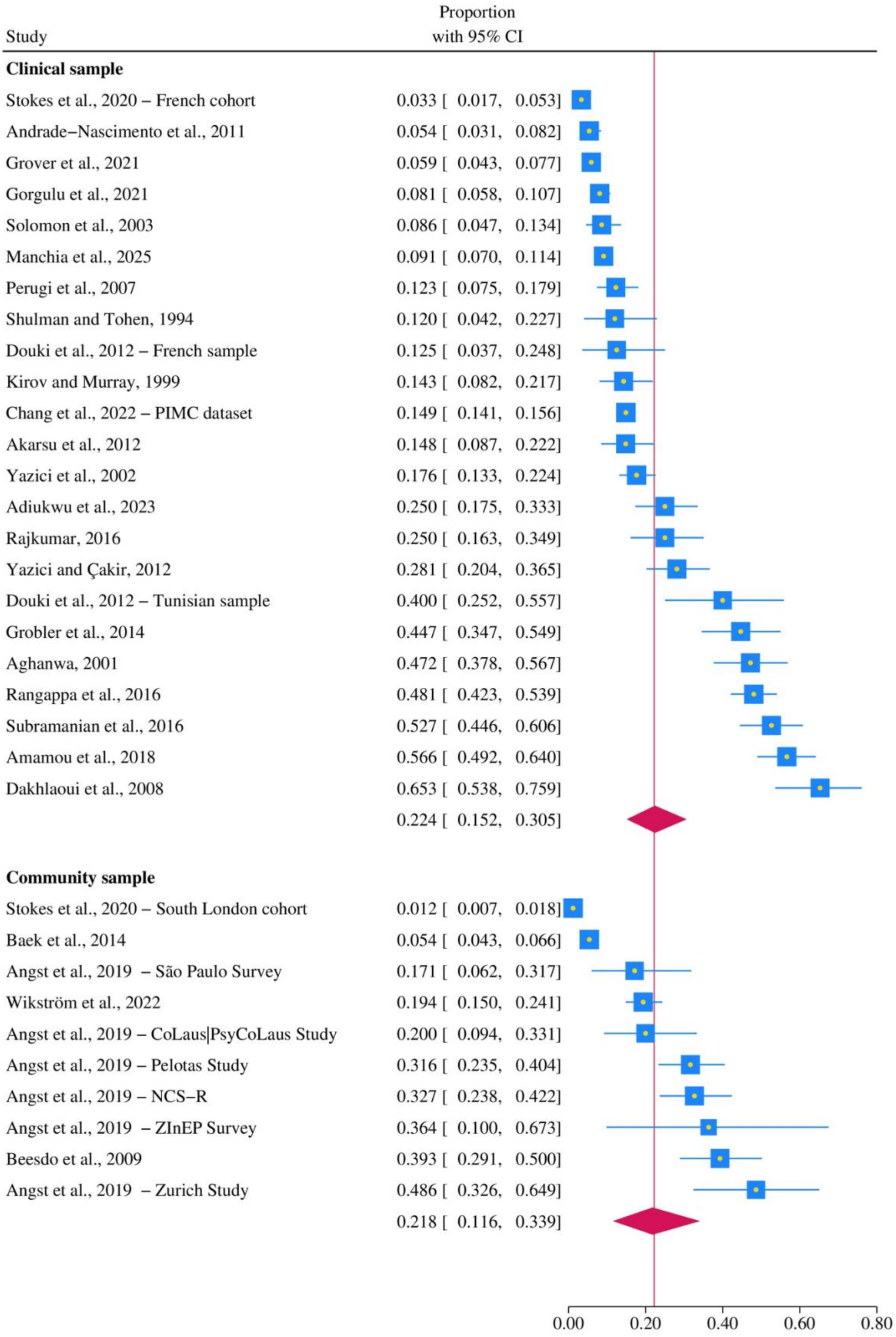

Abbreviations: **NCS-R**: National Comorbidity Survey Replication; **PIMC**: Psychiatric Inpatients Medical Claim; **ZInEP**: Zürcher Impulsprogramm zur nachhaltigen Entwicklung der Psychiatrie.

Supplementary Figure 2. Subgroup analysis according to geographical area (by continent).

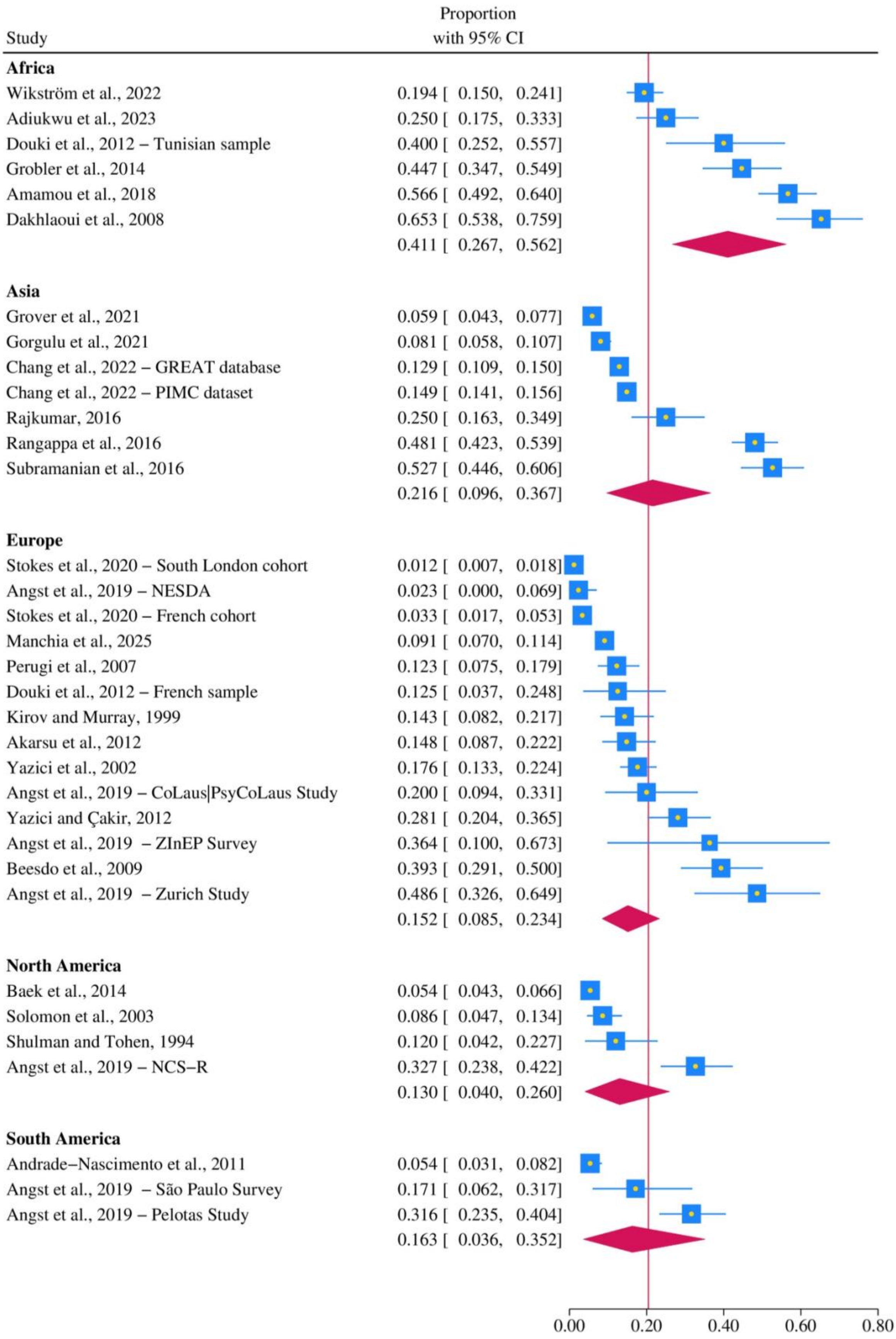

Abbreviations: **GREAT**: Genomic Research and Epidemiological Studies for Affective Disorders in Taiwan; **NCS-R**: National Comorbidity Survey Replication; **NESDA**: Netherlands Study of Depression and Anxiety; **PIMC**: Psychiatric Inpatients Medical Claim; **ZInEP**: Zürcher Impulsprogramm zur nachhaltigen Entwicklung der Psychiatrie.

Supplementary Figure 3. Subgroup analysis according to study quality.

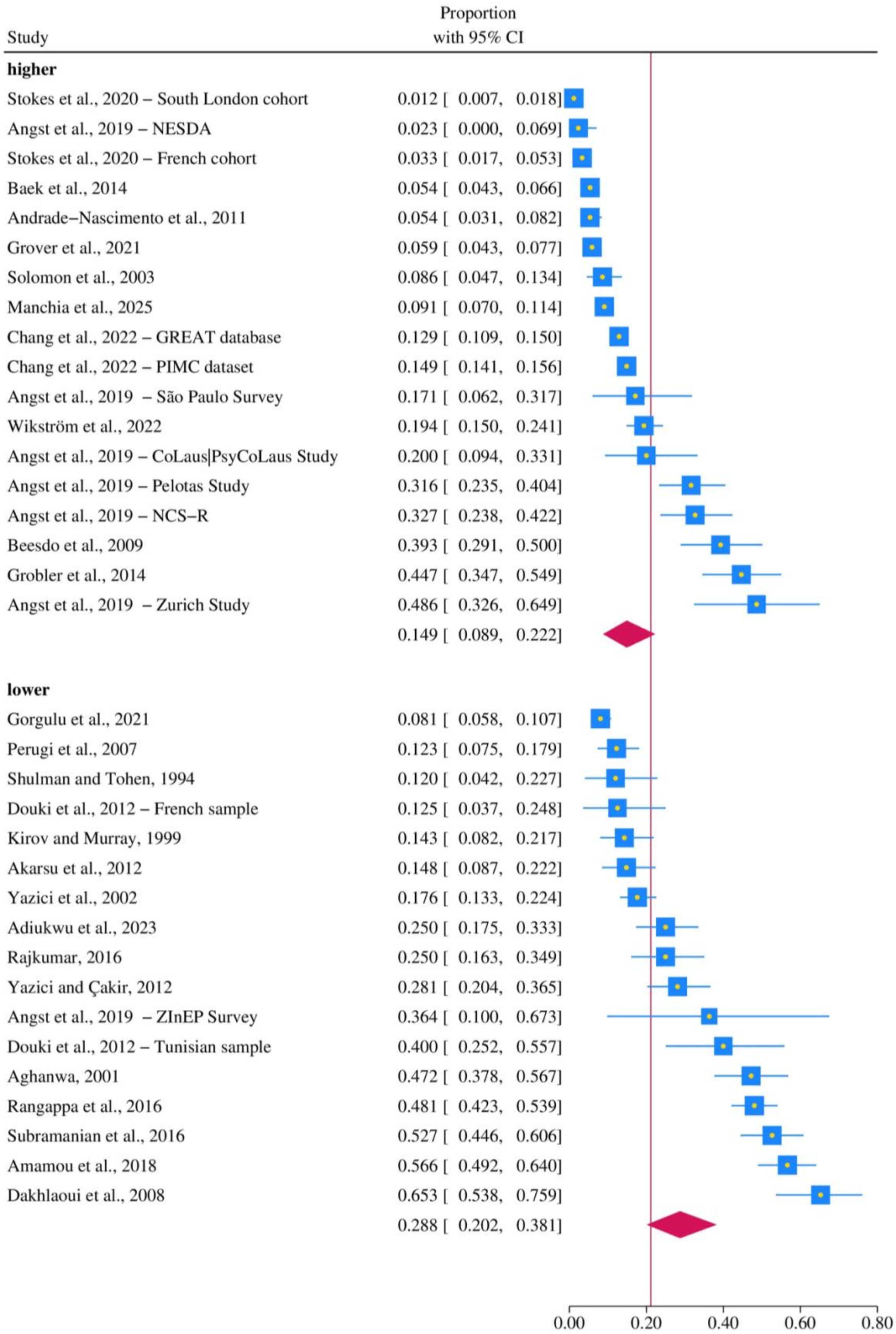

Abbreviations: **GREAT**: Genomic Research and Epidemiological Studies for Affective Disorders in Taiwan; **NCS-R**: National Comorbidity Survey Replication; **NESDA**: Netherlands Study of Depression and Anxiety; **PIMC**: Psychiatric Inpatients Medical Claim; **ZInEP**: Zürcher Impulsprogramm zur nachhaltigen Entwicklung der Psychiatrie.

Supplementary Figure 4. Subgroup analysis of studies requiring at least 1 episode to diagnose unipolar mania.

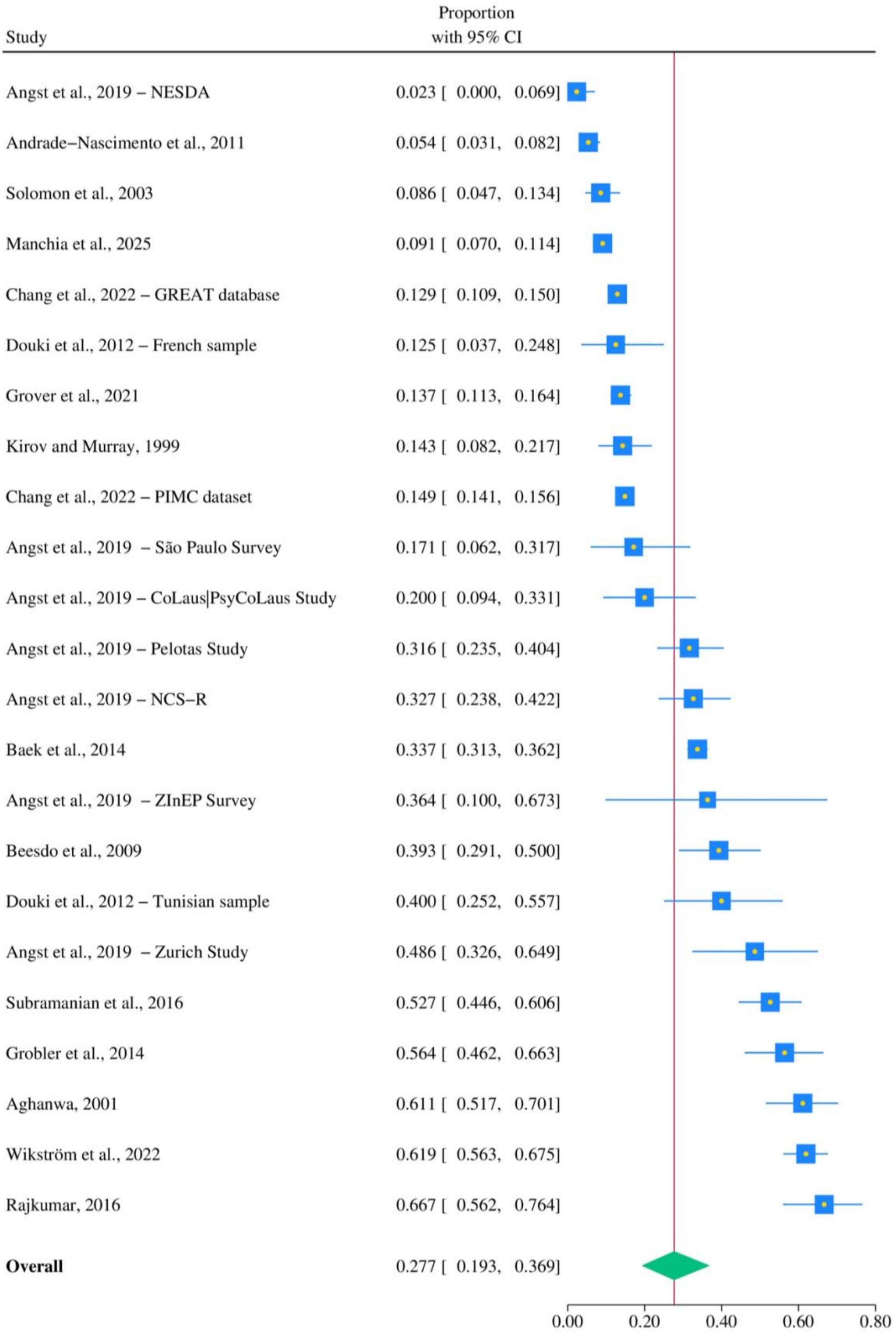

Abbreviations: **GREAT**: Genomic Research and Epidemiological Studies for Affective Disorders in Taiwan; **NCS-R**: National Comorbidity Survey Replication; **NESDA**: Netherlands Study of Depression and Anxiety; **PIMC**: Psychiatric Inpatients Medical Claim; **ZInEP**: Zürcher Impulsprogramm zur nachhaltigen Entwicklung der Psychiatrie.

Supplementary Figure 5. Subgroup analysis of studies requiring at least 2 episodes to diagnose unipolar mania.

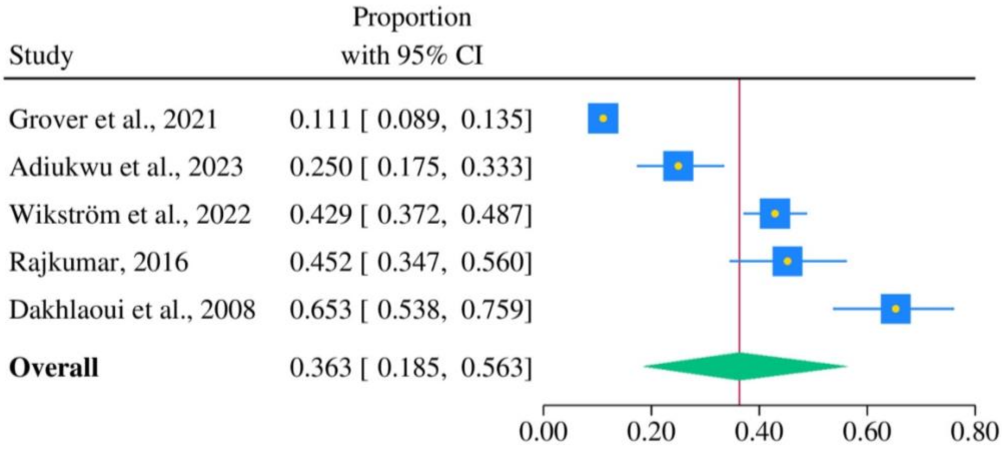

Supplementary Figure 6. Subgroup analysis of studies requiring at least 3 episodes to diagnose unipolar mania.

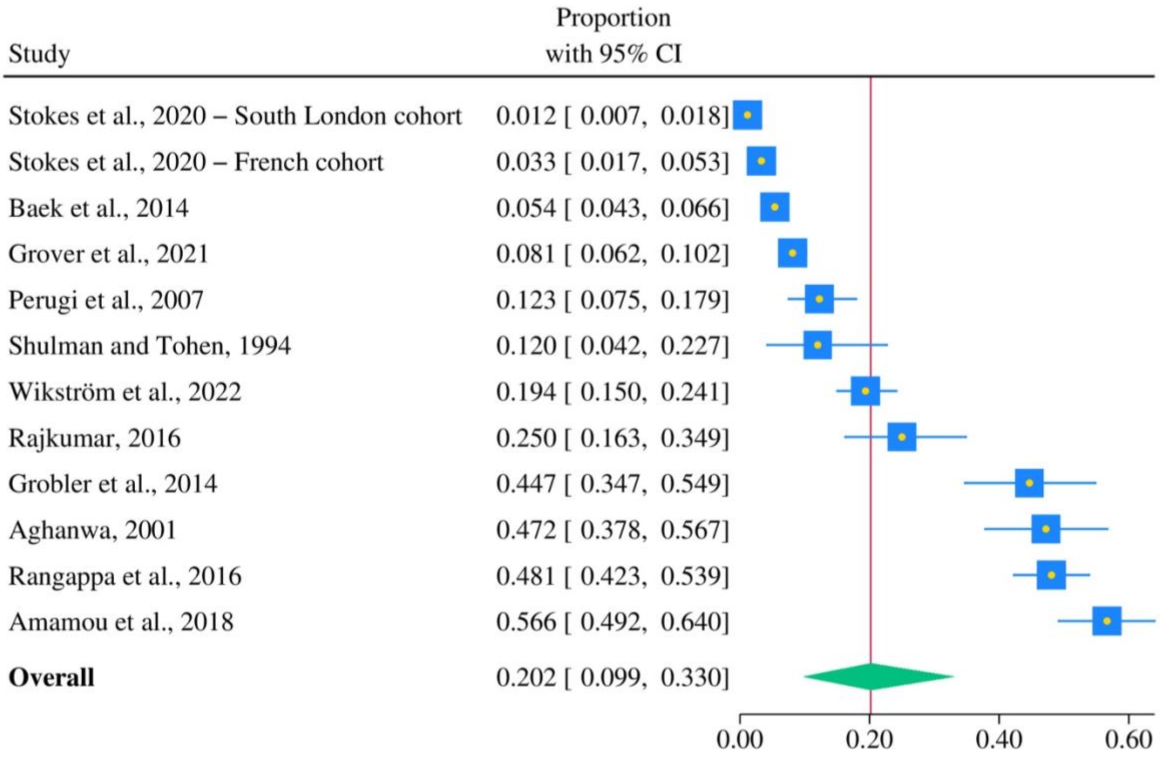

Supplementary Figure 7. Subgroup analysis of studies requiring at least 4 episodes to diagnose unipolar mania.

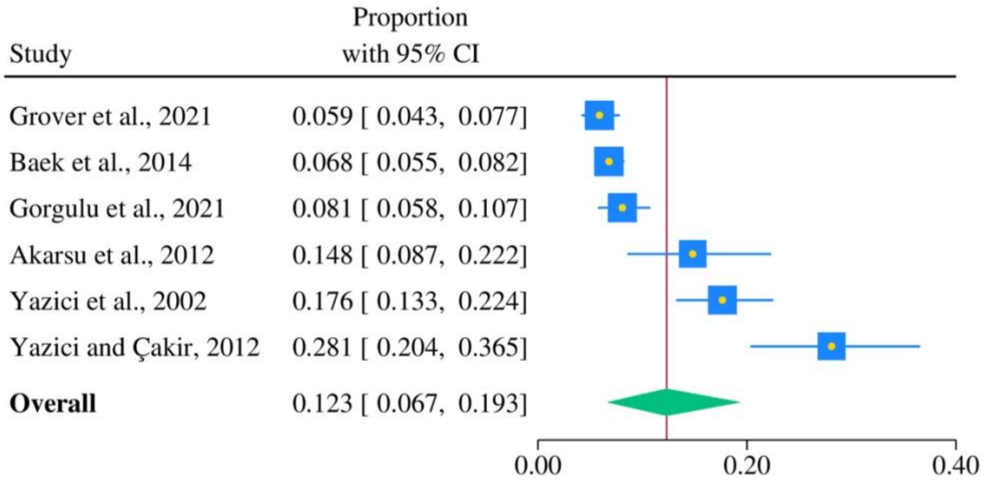

Supplementary Figure 8. Subgroup analysis of studies requiring none or minimal length of observation.

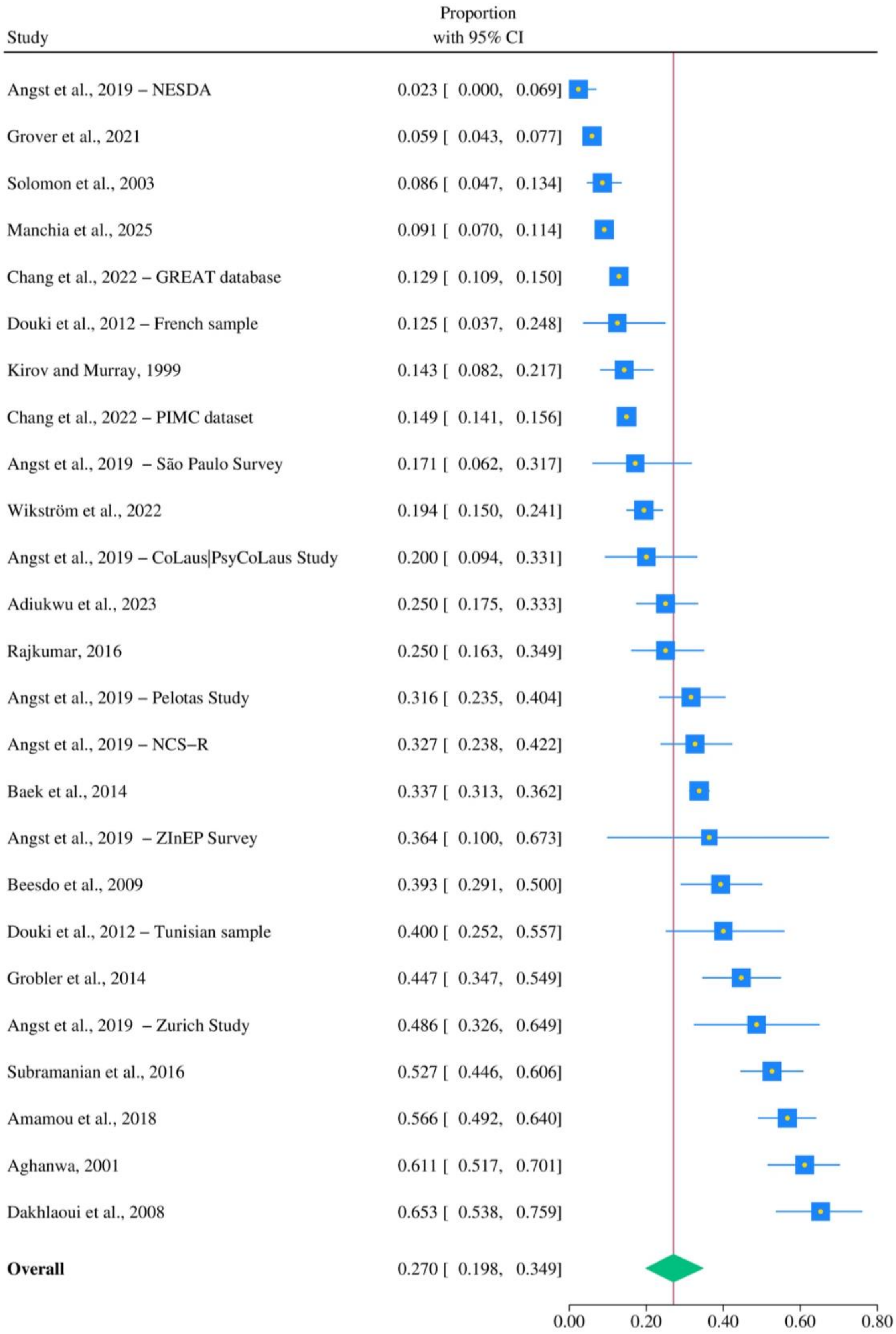

Abbreviations: **GREAT**: Genomic Research and Epidemiological Studies for Affective Disorders in Taiwan; **NCS-R**: National Comorbidity Survey Replication; **NESDA**: Netherlands Study of Depression and Anxiety; **PIMC**: Psychiatric Inpatients Medical Claim; **ZInEP**: Zürcher Impulsprogramm zur nachhaltigen Entwicklung der Psychiatrie.

Supplementary Figure 9. Subgroup analysis of studies requiring at least 4 or 5 years of observation.

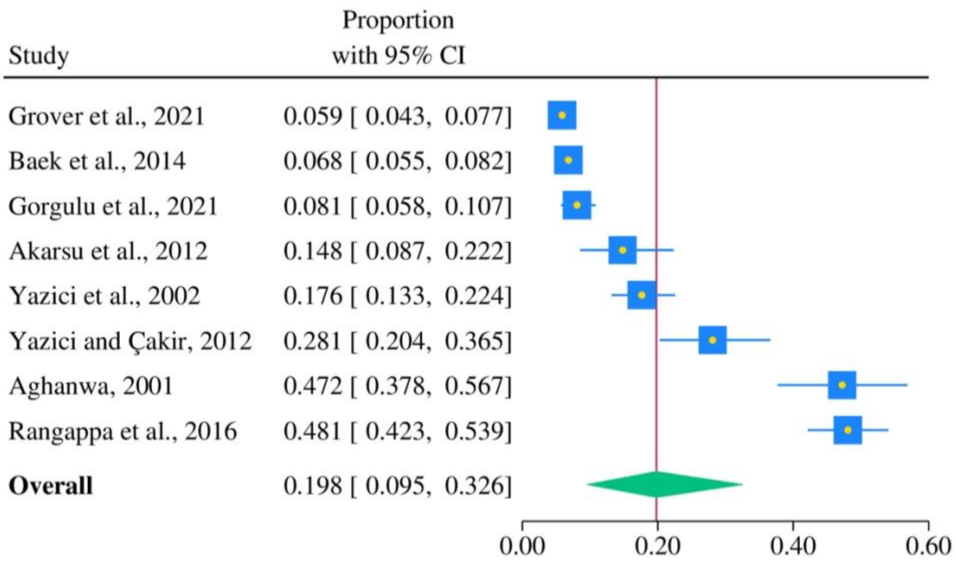

Supplementary Figure 10. Subgroup analysis of studies requiring at least 10 years of observation.

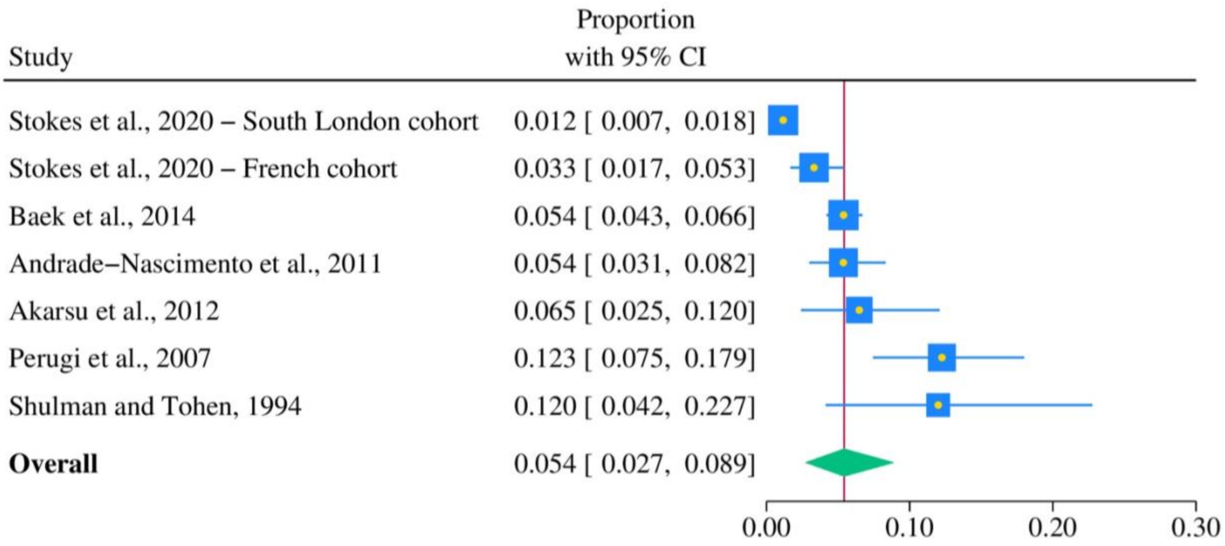

Supplement: Bartoli et al. supplementary material [file S2045796026100791sup001.pdf]
